# High diagnostic accuracy of chronic facial pain conditions via remote consultation

**DOI:** 10.22514/jofph.2025.028

**Published:** 2025-06-12

**Authors:** Tathyane H. N. Teshima, Roddy S. McMillan

**Affiliations:** ^1^Department of Oral Medicine, UCL Eastman Dental Institute, WC1E 6DE London, UK; ^2^Department of Oral Medicine and Facial Pain, Royal National ENT & Eastman Dental Hospitals, University College London Hospitals NHS Foundation Trust, WC1E 6DG London, UK

**Keywords:** Facial pain, Telephone consultation, Diagnosis, Sensitivity

## Abstract

**Background**: This study aims to evaluate the diagnostic accuracy of chronic facial pain conditions through telephone consultations. **Methods**: Retrospective cross-sectional service evaluation of diagnosis during remote against face-to-face consultations were compared, based on international classification of orofacial pain. Clinical data from consecutive new patients were assessed from March–May 2020 at University College London Hospitals (UCLH)-Eastman Dental Hospital, UK, reviewed independently by two specialists. Exclusion criteria included non-English speakers, patients unable to engage in consultation, unrelated pain diagnoses at assessment and inadequate documentation. Accuracy test of sensitivity, specificity and predictive values were employed. **Results**: A total of 93 patients were included, and 6 orofacial pain diagnoses met. Nearly 25% had at least one imaging test, however none demonstrated underlying cause or change of diagnosis. Overall diagnostic accuracy was 97.85% with 100% sensitivity and specificity with perfect inter-rater agreement (kappa = 1). Only persistent idiopathic facial pain and post-traumatic neuropathic pain had reduced accuracy (98.9%) and positive predictive value (75% and 50% respectively), being 100% for all other conditions. **Conclusions**: This study showed high diagnostic accuracy for diagnosing facial pain remotely, corroborating previous reports. Further investigations and physical examinations have not changed the diagnosis or management plan. This study evidenced that remote structured consultations represent a safe strategy for accurate facial pain diagnosis that may improve clinical efficiency.

## 1. Introduction

The alternative clinical assessment approach of using telephone consultations (TC) emerged during the COVID-19 pandemic restrictions in 2020. Due to constraints on travel and face-to-face clinical care, the facial pain (FP) outpatient service at the Royal National Ear Nose and Throat (ENT) & Eastman Dental Hospitals, UK was temporarily transformed into an exclusively remote service on the empirical assumption that a minority of cases would warrant clinical examination to confirm diagnosis. One year after the onset of restrictions, the service was switched to a mixed approach of remote and physical consultations to meet government restrictions and address patients’ concerns. This provided a unique opportunity to analyze the accuracy of FP diagnoses provided remotely without a physical assessment. This study aimed to achieve this by comparing the diagnosis of patients assessed via TC and again after clinical examination, using their face-to-face clinical assessment as the gold standard test.

The concept of remote dentistry has evolved from telemedicine projects (communication technologies and electronic systems) for over 30 years, aiming to provide quality care to patients in a cost-effective service [1]. More recently, systematic reviews reported that many studies could demonstrate the benefit of telephone consultations across primary care in reducing workload and in increasing patient satisfaction, with a potential reduction for the need of physical examination in half of the cases [2, 3]. These reviews have, however, reported inconsistencies and lack of strong evidence regarding the safety and quality of care in these studies.

This service evaluation aimed to assess the diagnostic value and effectiveness of remote facial pain consultations. The specific aim was to estimate the diagnostic accuracy of the initial telephone consultation against face-to-face review as the reference standard for new facial pain patients. We hypothesized that remote consultations are an accurate approach for diagnosing facial pain conditions if the pain history and clinical information is assessed appropriately. We also aimed to identify any factors that may reduce the effectiveness of remote consultations to enable changes in clinical practice which improve patient care.

## 2. Materials and methods

Retrospective cross-sectional analysis compared the initial diagnosis made during the remote facial pain consultations against the diagnosis confirmed by a follow-up face-to-face review, which was employed as the reference standard. Clinical notes of consecutive new patients assessed via telephone in the FP outpatient service from the 30th of March 2020 until the 28th of May 2020 were fully available and were independently collected by the reviewers from the hospital system for the study. Only patients that have been followed up with a face-to-face clinical visit until the time of analysis up to September 2021 were included. Each patient had the same time allocated for appointments. Accepted referrals to the facial pain service include patients who fulfil the criteria of chronic orofacial pain and having dental or ENT pathology excluded prior to referral; referrals for patients with acute facial pain (less than 3 months duration), dental or ENT related pain or primary headache disorders are not accepted. Estimation of diagnostic accuracy, sensitivity, specificity and inter-rater agreement of the index test were calculated.

Exclusion criteria included non-fluent English speakers who required an interpreter for the consultation and patients unable to engage in the consultation (*e.g.*, patients with learning disabilities or who refused to complete the encounter). Although such patients are normally seen in our routine facial pain face-to-face clinics, these factors could potentially limit the amount of information that could be captured remotely. Additionally, at the time of the study there was no remote access interpreter service available. Further exclusion reasons included patients with unrelated pain diagnoses (*e.g.*, primary diagnoses of dental related pain, acute pain, headache and migraine). This exclusion criterium was included as our service would not agree to see such patients but would instead forward such referrals to other specialists (dental department or headache specialist) or a local medical or dental practitioner. Cases with absence of facial pain symptoms at initial assessment and inadequate or missing clinical data which precludes a diagnosis being made were also excluded. In order to allow for an accurate diagnosis, a detailed pain history was collated using a standardised proforma which included: onset, site, duration/frequency, pain character, radiation of pain, presence of autonomic/migraine features, exacerbating/alleviating factors, severity, past and current pain treatments and medical history and biopsychosocial background (**Supplementary material 1,2**).

Diagnostic definitions were based on the current International Classification of Orofacial Pain (ICOP) [4] and included facial pain conditions regularly assessed and managed such as temporomandibular disorders (TMD), persistent idiopathic facial pain (PIFP), trigeminal neuralgia (TN), burning mouth syndrome/oral dysaesthesia (BMS/OD), persistent idiopathic dentoalveolar pain (PIDP) and post-traumatic trigeminal neuropathic pain (TNP). Burning mouth syndrome and oral dysaesthesia, which have almost identical symptomatology, could reasonably be considered synonymous. Data collection captured clinical data from new patients to the facial pain service who received an assessment and diagnosis at the initial telephone consultation and the assessment and diagnosis provided at the following first face-to-face appointment (regardless of number of previous telephone consultations), as well as details of any additional investigations. All diagnostic synonyms were accepted according to the current classification of orofacial pain [4].

The accuracy of the facial pain diagnoses for the remote and consequent face-to-face clinical consultations were assessed independently by 2 FP clinicians. The assessing clinicians included a range of oral medicine and facial pain specialty doctors, oral medicine specialty trainees and oral medicine consultants (specialists), all appropriately trained for working within the facial pain service. In the event of a dispute, a third clinician, an oral medicine consultant, would be asked to decide on the accuracy of any given diagnosis. In the event of inadequate documentation to permit confirmation of an FP diagnosis, the case would be excluded from the analysis.

## 3. Results

A total of 212 new patients were identified across all available facial pain clinics within the included timeframe. After screening, 93 patients were deemed eligible and were included in the study (summary in STARD (Standards for Reporting Diagnostic accuracy studies) flowchart in Fig. 1). The cases excluded after the initial screening were mostly due to the symptom being unrelated to a FP diagnosis, such as suspected oral mucosal disease, headaches or migraine diagnoses (n = 24), followed by the assessment not being an initial “new patient” consultation (n = 3). Further to the first screening, a second cohort was excluded due to no attendance to or no arrangement of a follow-up appointment (n = 92/185, of which 35 did not attend a booked follow-up appointment).

**Fig. 1.** Standard for reporting diagnostic accuracy (STARD) flowchart. Illustration of the inclusion process of eligible patients into the study. A total of 212 patients were identified initially, resulting in 93 cases included in analysis after exclusion due to lack of eligible symptoms, not being a new patient to the service, insufficient documentation of telephone consultation or no face-to-face follow up as index test comparator. Only 2 patients had a change of diagnosis as index test negative, whereas 91 had no change to the initial TC diagnosis. TMD: temporomandibular disorders; PIFP: persistent idiopathic facial pain; PIDP: persistent idiopathic dentoalveolar pain; TNP: trigeminal neuropathic pain; BMS: burning mouth syndrome; TN: trigeminal neuralgia.

The average age of eligible patients assessed ranged from 20 to 82 years old (mean = 52.6), and most patients were female (79 compared to 14). Regarding diagnosis, 58 cases were diagnosed as pain-related TMD (chronic primary myofascial pain), followed by 24 diagnosed with TNP, then less frequently PIFP (n = 4), BMS/OD (n = 4), PIDP (n = 2) and TN (n = 1) (Table 1). No other TMD related pain with an arthritic or articular cause were identified in our cohort, although they were considered in all new patient assessments.

**Table 1.** Summary of baseline clinical data from eligible patients related to type of diagnosis and requested imaging tests.

Number of patients included from screening		N = 93 (119 excluded)	Diagnosis—N					
			Pain-related TMD	TNP	PIFP	BMS/OD	PIDP	TN
Gender								
	F	79	51	21	2	4	1	0
	M	14	7	3	2	0	1	1
Total			58	24	4	4	2	1
Age range N (median)		20–82 (55)						
	F		20–78	34–82	67–70	50–81	75	N/A
	M		37–65	42–74	57–61	N/A	61	74
Further imaging (total)		23	15	6	0	1	0	1
	Ultrasound		3	1	0	1	0	0
	Head or TMJ magnetic resonance		12	3	0	0	0	1
	CBCT		0	2	0	0	0	0

*TMD: temporomandibular disorders; TNP: post-traumatic trigeminal neuropathic pain; PIFP: persistent idiopathic facial pain; BMS/OD: burning mouth syndrome/oral dysesthesia; PIDP: persistent idiopathic dentoalveolar pain; TN: trigeminal neuralgia; F: female; M: male; TMJ: temporomandibular joint; CBCT: Cone Beam Computed Tomography; N/A: Not applicable.*

Most patients were diagnosed with pain-related TMD (58/93), followed by TNP (24/93) and other diagnoses, affecting more women than men. Further imaging tests were requested to a total of 23 patients in this cohort (26%), most commonly for TMD symptoms. All cases that required further investigations to be conducted were analysed (Table 1). A total of 23 patients (24.7%) had at least one imaging test requested, however none of them resulted in a change of diagnosis. Most scans were requested for TMD (n = 15/23), followed by TNP (n = 6/23), and equally reported for BMS/OD and TN (n = 1/23 each). The type of imaging tests requested included magnetic resonance (MR) of the head, MR temporomandibular joint (TMJ), ultrasound of salivary glands/neck (US) and Cone Beam Computed Tomography (CBCT) for ruling out dental pathology. Orthopantomograms were only requested prior to internal referral to the dental specialties for a second opinion as part of a local care pathway agreement. Prior to referral to our service, all patients are expected to have a dental cause for the pain excluded by a primary care dental surgeon. The reasons for imaging request were reported as: for reassurance to patients, presence of allodynia/neuropathy, abnormalities of mandibular function during examination (jaw deviation, crepitus), history of head and neck trauma, pathology exclusion for functional neurological disorder, previous temporomandibular joint surgery, history of arthritis and report of new or progressive pain symptoms. About a third of these patients (n = 9/24) had one of these reasons to have had the investigation, whereas the remaining 15 patients were either for reassurance or no specific reason was documented.

The diagnostic accuracy of facial pain conditions for new patients via telephone consultation was analysed, considering the assessment during the consequent face-to-face appointment as the reference standard. This was summarised in Fig. 2. The overall diagnostic accuracy resulted in 97.8% accuracy (91/93 cases accurate), where only PIFP and PIDP showed inferior accuracy (1/4 and 1/2 cases were inaccurate respectively) compared to all other diagnoses that presented with 100% diagnostic accuracy. The two cases that had their diagnoses changed after face-to-face consultation were initially diagnosed as PIFP and PIDP (one each): one changed from PIDP to PIFP as there was poorly localised pain confirmed with examination, and another from PIFP to TMD with post-traumatic trigeminal neuropathic pain in another case after reviewing the pain history and noticing the presence of muscular pain and localised allodynia on examination. No additional imaging was requested for either case. Diagnostic accuracy, sensitivity, specificity and predictive values (positive and negative) were calculated as 100% for all conditions, with exception of PIFP and PIDP that showed reduced diagnostic accuracy of 98.9%, specificity (99%) and positive predictive value (75% and 50% respectively). There was perfect inter-rater agreement noted (kappa = 1).

**Fig. 2.** Summary of FP diagnostic accuracy. The overall diagnostic accuracy was 97.8% as accounted for reduced accuracy of PIFP and PIDP that showed 98.9% each. All other conditions resulted in 100% diagnostic accuracy in this study. TMD: temporomandibular disorders; TNP: post-traumatic trigeminal neuropathic pain; PIFP: persistent idiopathic facial pain; BMS/OD: burning mouth syndrome/oral dysesthesia; PIDP: persistent idiopathic dentoalveolar pain; TN: trigeminal neuralgia.

## 4. Discussion

Our results showed a positive diagnostic accuracy of orofacial pain conditions diagnosed via remote consultation, particularly evident for pain-related temporomandibular disorder. Importantly, we noted that further brain, neck or jaw imaging resulted in no difference in the diagnostic decision, and there was no indication to refer for surgical opinion or need of any surgical procedures in our cohort. Most of these imaging cases were to rule out underlying pathology for pain-related TMD, and none found any pathological cause for the pain symptoms. This is in keeping with recent guidelines on the management of temporomandibular disorders, where routine imaging specifically for the diagnosis of pain-related TMD is currently not supported, including panoramic radiographs, CBCT, MR and ultrasound [5, 6]. Nonetheless, the use of imaging for diagnosing TMD conditions and excluding underlying arthritic or articular pathologies is still performed on a case-by-case approach, where imaging would be indicated especially in the presence of any significant restrictions in normal jaw movements, atypical signs or symptoms or history of trauma [7].

A detailed retrospective study performed in the US with data collected around the same time as this study also demonstrated a high proportion of 78.8% of new cases diagnosed via telephone or videoconference appointment remained the same after the following face-to-face visit [8]. The potential reasons for the reduced diagnostic accuracy compared to our cohort was not particularly clear from the reported study. Patients were assessed in the orofacial pain unit in a general hospital, and possibly involved more general pain specialists, with less experience of FP when compared to our tertiary unit. The study had comparable results in terms of conditions diagnosed, diagnostic classification used and had similar proportions of diagnoses. Interestingly, there was no difference in diagnostic accuracy when comparing telephone and video consultation, which may suggest no additional benefit of video over telephone consultations. Additional imaging was requested for 14% of patients compared to 26% in our cohort with no information whether there was change of diagnosis after imaging.

Another study from Brazil focused on analysing telediagnosis of temporomandibular disorders of 61 patients [9], reporting this as a feasible approach with good to low levels of inter-rater agreement for diagnosis, including moderate agreement for myofascial pain. A lower sensitivity rate of 78% for myofascial pain was reported compared to our study, where the only diagnostic criteria used was the Symptom Questionnaire – diagnostic criteria for temporomandibular disorders (SQ—DC/TMD). There was no clear information provided about the assessors involved in this study, although training sessions and convenience sample analysis were reported prior to recruiting patients. Another important distinction was the time between teleconsultation and face-to-face assessment, which was only 20 minutes as under ideal research circumstances, compared to our pragmatic approach in routine clinical practice. It was also not clear whether the Axis II protocol or similar tool that includes risk factor assessment was utilised in the diagnosis and management plan.

A large multicentre non-randomised study from 2012 from Spanish primary care centres and an oral and maxillofacial surgery unit investigated the effectiveness of telemedicine in selection, diagnosis and treatment of temporomandibular disorders [10]. This included the diagnosis of myofascial pain and/or internal temporomandibular joint derangement and other arthritic pathologies. Of the 342 patients included, 10% presented with temporomandibular joint pathology and required maxillofacial surgery and the majority had high resolution of condition via remote consultations. All patients received the same protocol of baseline imaging investigations with panoramic radiograph and tomography under research conditions, in contrast to our pragmatic approach. The interobserver agreement also seemed high (89%) between a mix of primary dental practice and hospital staff members from the oral maxillofacial surgery unit. It was unclear which diagnostic criteria were utilised, and the ratio of patients assessed in each centre. Another important finding was the notable reduction in waiting time for treatment, where telemedicine allowed the mean time elapsed to treatment from 78.6 to 2.3 days, which is a valuable benefit for the clinical activity of high demand centres.

The diagnostic accuracy of remote consultations for some medical specialties have been evaluated since the pre-onset of COVID-19 pandemic. These previous studies from rheumatology, dermatology, psychiatry and breast cancer clinics reported a range from 70–89% diagnostic accuracy, with generally high predictive values [11, 12, 13, 14, 15]. A particular study from an oral and maxillofacial surgery unit in the UK has reported the diagnostic accuracy of telephone consultations during COVID-19 restrictions [16]. Their results demonstrated a subgroup analysis of facial pain diagnosis, which had the least need for face-to-face consultation (about 45%), the highest rate of discharges compared to other conditions included in the study (55%) and no case required imaging or surgical procedure. This study however included a small number of cases compared to the included group (6 out of 337 patients in total), did not specify which facial pain conditions were included and did not distinguish between new or long-term follow-up patients, and hence cannot be comparable to our findings.

Another useful finding from their study was about understanding patient experience and satisfaction when having remote consultations compared to standard face-to-face. This is of particular importance considering the cost of delivering healthcare and moving towards a more patient-centred approach to care. Their survey reported that overall, most patients in the unit strongly or moderately preferred a remote consultation compared to face-to-face (59% and 12.5% respectively, n = 337), with a similar picture for the small cohort of facial pain patients (66%, n = 6). Other positive findings included time, effort and cost-related benefits of remote consultations, with the opposite outcome for hearing difficulties, technical issues and language barriers as potential concerns of this modality. Another study measured the time and cost effectiveness of these consultations in comparison to presential ones, reducing the mean cost of lost working hours/patient in half (32 to 16 h) of over 300 patients [10].

A review in 2021 analysing studies in patient evaluation of teleconsultations showed 4 studies with positive patient satisfaction and adherence to follow up via remote consultations diagnosed with chronic facial pain [3]. Another small survey study from Australia also demonstrated good patient satisfaction of the quality of remote consultations for managing chronic facial pain [17]. Those positive patients experience reports were in accordance with a hospital-wide standardised service evaluation conducted by the authors’ unit, which showed that more than 68% (n = 78) of patients assessed by the facial pain department preferred remote consultation, mainly due to time and cost-related efficiency (data not published as it was part of local service evaluation). One of the main concerns from clinicians and patients in this survey was regarding the diagnostic accuracy and safety, with need of physical examination (43.5%) and risk of missed malignancy (10.9%), which has been addressed by our findings.

Identifying that imaging tests were not relevant for the confirmation of facial pain diagnoses was another important finding of this study. Commonly, patients present with major concern of underlying pathology to explain pain symptoms and imaging tests may be requested to enhance reassurance without any obvious clinical indication, which could impact upon imaging resources and unnecessarily expose patients to ionising radiation. In this study, most patients who had any imaging had no clear reason documented for such a request (15/24). Undoubtably, imaging investigation is often indicated to identify any trigeminal nerve damage by pathology, arthritis and neoplastic conditions, particularly if there is suspected neuropathic pain or trigeminal neuralgia. However, consideration for imaging should be decided following an appropriate assessment and when the outcome of the scan could potentially change the diagnosis or clinical management [7, 18].

With the advances of artificial intelligence (AI), some studies have reported the usefulness of the technology in facilitating self-diagnosis and aiding appropriate referral, although still with limitations. A study in 2018 reported the accuracy of a predictive model to diagnose jaw pain using informatics technology comparing medical records of genuine cases and rare TMD-mimicking cases [19]. The predictive performance of the model resulted in 69% sensitivity and 99.3% specificity in predicting TMD-mimicking conditions, however the descriptors of pain and associated medical history incorporated in the automated models have a major impact on the result and further studies will be required if AI is to be considered as a diagnostic approach. Similarly, a systematic review in screening tools for trigeminal neuralgia demonstrated that a proposed learning machine was still unable to appropriately diagnose the condition based on clinical records [20]. A scoping review from 2023 looked at different modalities of telemedicine for pain management derived from multiple conditions (cancer, orofacial pain, musculoskeletal and unspecified chronic pain) highlighted the heterogeneity of tools and outcome measures, varying according to aims and available resources. It, however, concluded that the overall results were beneficial for patient satisfaction, pain improvement and access to care [21].

This study provided valuable evidence of a high diagnostic accuracy and safe approach when performing remote assessment of chronic facial pain. This has the potential to open avenues to establish alternative remote access chronic facial pain services with the associated significant potential cost and time benefits for both patients and care providers. However, this study being a retrospective analysis of patients referred to a tertiary specialist centre limiting the applicability of the outcomes to the wider cohort of facial pain patients, especially for the most common conditions such as TMD. Another constraint of the study is that the available data was acquired at a tertiary care centre, where patients are assessed by a specialist team using a comprehensive and standardised pain history assessment. This makes it less likely to be generalisable and replicated in non-specialist units, given that appropriate staff training and specialist support would be required. This study was limited to be designed as cross-sectional in view of the service returning to exclusive face-to-face clinics for new patients once pandemic restrictions were lifted, potentially resulting in causal inference. There may have also been information bias in the process of diagnosis. We highlight the importance of having a low threshold for imaging on a case-by-case basis for cases of trigeminal neuralgia, trigeminal neuropathy or when any atypical pain features are present. Moreover, incorporating validated screening questionnaires is a helpful resource to indicate specific chronic facial pain diagnoses such as the 3 question TMD screener (3Q/TMD) to enhance the diagnostic process and appropriate referral and management [22].

## 5. Conclusions

This study does not indicate that a face-to-face service should be fully replaced by a remote access system, but it does demonstrate that a remote consultation can be safely and effectively applied in the vast majority of cases. Non-verbal communication and face-to-face interaction can be considered useful as part of a holistic assessment and management planning process. Despite its limitations, the outcomes do appear to support the integration of telephone consultations into chronic facial pain practice as a way of safely improving clinical effectiveness and patient access.

## Supplementary Material

Supplementary material associated with this article can be found, in the online version, at https://files.jofph.com/files/article/1933037380553326592/attachment/Supplementary%20material.docx.


