# Supplementary material for: High diagnostic accuracy of chronic facial pain conditions via remote consultation
Source: J Oral Facial Pain Headache. 2025 Jun 12;39(2):94–100. doi: 10.22514/jofph.2025.028 (PMC12520444; doi:10.22514/jofph.2025.028)
Supplement: Supplementary file 1 [file Supplementary-material.docx]

Supplementary material 1

Facial Pain Assessment

**Seen: on their own/accompanied/English not first language/Interpreter (details: )**

| **Facial Pain First Visit** | | |
| --- | --- | --- |
| ***Date:*** | ***Consultant:*** | ***Hospital No:*** |
|  | | ***Name:*** |
| ***Referrer:*** | ***GP □ Dentist □ Specialist □***  ***Self □*** | ***Date of birth: M/F*** |
| **Care of:** | | |
| **History of presenting complaint:** | | |
|  | | |

**Pain history:**

| Timing: When did the pain first start: Date:  Weeks □ Months □ Year(s) □ |
| --- |
| **Did any event occur at the time of onset of pain?**  **No** □  **Yes** □ : Dental treatment □ endodontics □ oral surgery □ other dental procedures □  Trauma □ where?  History of: Operation □ Herpes zoster □ Illness □ Stress □ Other □ |
| **Circumstances around first attack**:  Acute □ Slow to develop □ Memorable □ cannot remember □ |
| **Severity of pain from Brief Pain Inventory questionnaire:**  average (/10) worst (/10) least (/10)  Has this become more intense recently: Yes □ No □  Is the pain: Continuous □ Episodic □  Has pain changed from episodic to continuous: Yes □ No □ |
| **If pain is continuous:**  **Varying intensity:** Yes □ No □  Duration of the intense periods of pain: Hours Days Weeks Months  Frequency of intense periods: Hourly Daily Weekly Monthly  Duration of the less intense periods of pain: Hours Days Weeks Months  Proportion of intense periods: %  **Severity of pain 0–10:** the intense period ( ) least intense period ( ) |
| **If pain is episodic:**  **How often** are these attacks, on average? Hourly □ Daily □ >15 days a month □ <15 days a month □  **Attack duration**: seconds □ minutes □ 1–4 hours □ 4–72 hours □ longer than 3 days □  **Do the attacks start**: suddenly □ slowly □ **Do attacks go down**: suddenly □ slowly □  **Remission periods?** Yes □ No □  **How long are they:** average: range: date onset last attack:  **Are they getting:** shorter □ longer □ no change □  Is the primary pain especially strong at specific times? Yes □ No □  if so, when? morning □ mid-day □ afternoon □ evening □ night, while asleep □  women: when menstruating □  For trigeminal neuralgia: % attacks evoked % spontaneous  Is each attack of pain: single stab □ a series of stabs □ continuous but saw tooth like □ |

**Which figure below best represents the primary pain during the past 4 weeks?** Add in times and pain severity


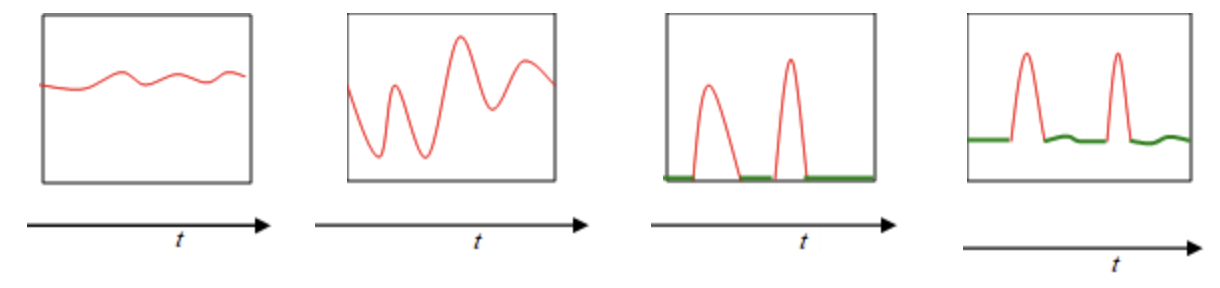


1) constant pain with minor variation

2) constant pain with major variation

3) attacks of acute pain, no pain in between single or multiple

4) attacks of acute pain, with continuous pain in between

| **Character: *do short McGill Pain Questionnaire (MPQ) below if language problems OR full MPQ, record actual expression*** | | | | | | | | | | | | | | |
| --- | --- | --- | --- | --- | --- | --- | --- | --- | --- | --- | --- | --- | --- | --- |
| Tiring/exhausting  Sickening  Fearful  Punishing-cruel  Piercing  Pain on touch  Burning/Hot  Pins and needles  Itchy  Electric–shock  Cold  Gnawing  Aching  Heavy  Tender  Splitting  Throbbing  Shooting  Stabbing  Sharp  Cramping  Numbness  Electric  **Other** | | | | | | | | | | | | | | |
| **MCGILL PAIN (MGP) QUESTIONNAIRE - only if adequate language** | | | | | | | | | | | | | | |
| Circle the words below that best described your current pain. If more than one pain label 1 & 2 | | | | | | | | | | | | | | |
| Use only one word in each group. | | | | | | | | | | | | | | |
| Leave out any group if the words are unsuitable. | | | | | | | | | | | | | | |
| 1  Flickering  Quivering  Pulsing  Throbbing  Beating  Pounding | | | 2  Jumping  Flashing  Shooting | | | | 3  Pricking  Boring  Drilling  Stabbing  Lancinating | | | | | 4  Sharp  Cutting  Lacerating | | |
| 5  Pinching  Pressing  Gnawing  Cramping  Crushing | | | 6  Tugging  Pulling  Wrenching | | | | 7  Hot  Burning  Scalding  Searing | | | | | 8  Tingling  Itchy  Smarting  Stinging | | |
| 9  Dull  Sore  Hurting  Aching  Heavy | | | 10  Tender  Taut  Rasping  Splitting | | | | 11  Tiring  Exhausting | | | | | 12  Sickening  Suffocating | | |
| 13  Fearful  Frightful  Terrifying | | | 14  Punishing  Gruelling  Cruel  Vicious  Killing | | | | 15  Wretched  Blinding | | | | | 16  Annoying  Troublesome  Miserable  Intense  Unbearable | | |
| 17  Spreading  Radiating  Penetrating  Piercing | | | 18  Tight  Numb  Drawing  Squeezing  Tearing | | | | 19  Cool  Cold  Freezing | | | | | 20  Nagging  Nauseating  Agonizing  Dreadful  Torturing | | |
| **Site**: Right□ Left□ Bilateral □/Intraoral □ Extraoral □/ Superficial □ Deep □  *Mark sites of pain with a X, areas of radiation, trigger points, loss sensation* | | | | | | | | | | | | | | |
|  | | | | | | | | | | | | | | |
| 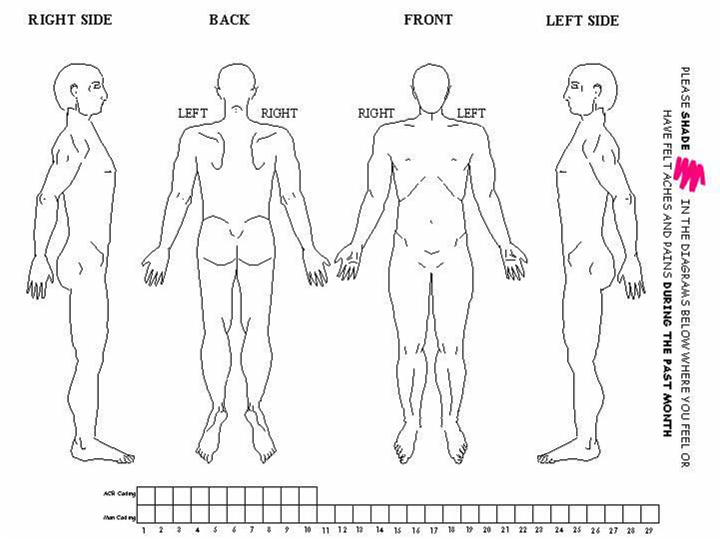rightleft  Any changes of site with time: Yes □ No □ | | | | | | | | | | | | | | |
| **Factors affecting pain** (tick only positive findings) | | | | | | | | | | | | | | |
| Factor | Provoking/  aggravating factors | | | | | Relieving | | | Factor | Provoking/  aggravating factors | | | | Relieving |
| Talking |  | | | | |  | | | Lying down |  | | | |  |
| Eating |  | | | | |  | | | Fatigue/tiredness |  | | | |  |
| Brushing teeth |  | | | | |  | | | Stress/tension |  | | | |  |
| Swallowing |  | | | | |  | | | Noise |  | | | |  |
| Shaving/washing/touch |  | | | | |  | | | Light |  | | | |  |
| Food: cold/hot/sweet |  | | | | |  | | | Smell |  | | | |  |
| Pressure on teeth/biting |  | | | | |  | | | Alcohol |  | | | |  |
| Chewing prolonged |  | | | | |  | | | Diet irregular |  | | | |  |
| Opening wide/yawning |  | | | | |  | | | Distraction |  | | | |  |
| Cold/wind/weather |  | | | | |  | | | None |  | | | |  |
| Warmth/weather |  | | | | |  | | | Medication |  | | | |  |
| Stooping/bending |  | | | | |  | | | Other |  | | | | |
| Bodily movement, increased physical activity |  | | | | |  | | |  |  |  |  |  |  |
| **Associated Factors** | | | | | | | | | | | | | | |
| **Factor** | | | | present | absent | | | **Factor** | | | present | | absent | |
| Dental problems | | | |  |  | | | Earache | | |  | |  | |
| Altered/poor taste | | | |  |  | | | Ringing in ears | | |  | |  | |
| Disturbed salivation | | | |  |  | | | Fullness in the ears | | |  | |  | |
| Altered/sensation/numbness | | | |  |  | | | Deafness side time | | |  | |  | |
| Bruxism/clenching | | | |  |  | | | Dizziness | | |  | |  | |
| Clicking joint/sticking | | | |  |  | | | Headaches | | |  | |  | |
| Inability to open wide | | | |  |  | | | Migraines +/− aura | | |  | |  | |
| Swelling mouth/face side | | | |  |  | | | Neck pain | | |  | |  | |
| Colour/redness site | | | |  |  | | | Back pain | | |  | |  | |
| Nasal stuffiness/runny | | | |  |  | | | Itchy skin | | |  | |  | |
| Post nasal drip | | | |  |  | | | Irritable bowel | | |  | |  | |
| Double/blurred vision | | | |  |  | | | Menstrual pain | | |  | |  | |
| Tearing/dryness | | | |  |  | | | Fatigue/loss of strength | | |  | |  | |
| Visual disturbances | | | |  |  | | | Impaired concentration | | |  | |  | |
| Eye redness | | | |  |  | | | Nausea/vomiting | | |  | |  | |
| Oedema eyelid/miosis | | | |  |  | | | Reduced appetite | | |  | |  | |
| Sweating | | | |  |  | | | Stress | | |  | |  | |
| When in pain restless/still/neither | | | |  |  | | | Other | | |  | |  | |
| **Sleep: Do you have sleep issues?** due to face pain □ due to other causes □  Have trouble falling asleep: Not at all □ Some of the time □ All the time □  Wake up several times per night: Not at all □ Some of the time □ All the time □  H Have trouble staying asleep: Not at all □ Some of the time □ All the time □  Wake up after your usual amount of sleep feeling tired and worn out:  Not at all □ Some of the time □ All the time □  Do you wake up early: No □ Yes □ | | | | | | | | | | | | | | |
| **Migraines/headaches present?** Yes □ No □  **if yes, provide more details if relevant:**  Site: character:  Age started: Frequency:  Duration of attacks: disabling headache (one day) □  Nausea □ Vomiting □ Photophobia □ Phonophobia □ Osmophobia □  Aura: Yes □ No □ Type:  **History of:**  Chronic fatigue syndrome □ Fibromylagia □ Chronic widespread pain □ | | | | | | | | | | | | | | |
| Past Treatments **Previous consultations:**  General practitioner□ Dentist □ Dental Specialist □ Oral surgeon □ Rheumatologist □  Ear Nose and Throat surgeon □ Neurosurgeon □ Neurologist □ Physician □  Psychiatrist □ Psychologist □ Pain specialist □ Counsellor □  Other □  **Current specialists being seen**:  **Treatment/Surgery:**  Splints □ TMJ injection □ Arthroscopy □ TMJ surgery □ Other surgery □  Restorative □ Endodontics □ Extraction □ Oral surgery □  Physiotherapy □ Osteopathy □ Acupuncture □ Low intensity laser □  Transcutaneous electrical nerve stimulation □  Homeopathy □ Hypnosis □ Chiropractic treatment □ Cognitive behavioral therapy□ | | | | | | | | | | | | | | |
| **Drugs** | | **Name: dates used, maximum dose, effectiveness and side effects (SE)** | | | | | | | | | | | | |
| Analgesic | |  | | | | | | | | | | | | |
| Indomethacin | |  | | | | | | | | | | | | |
| Antibiotic | |  | | | | | | | | | | | | |
| Tranquiliser | |  | | | | | | | | | | | | |
| Antidepressant | |  | | | | | | | | | | | | |
| Anticonvulsant | |  | | | | | | | | | | | | |
| Other | |  | | | | | | | | | | | | |

# Relevant previous medical history (see also separate medical sheet)

| **Trigeminal Surgery and effect** | **Date** | **Place** |
| --- | --- | --- |
| Peripheral |  |  |
| Glycerol radiofrequency thermocoagulation/Micro-compression |  |  |
| Gamma knife |  |  |
| Microvascular decompression/percutaneous stereotactic rhizotomy |  |  |
| Diagnosis (if any given by the patient)/Beliefs:  **Effect of Pain**  What changes have occurred in your life as a result of the pain?  During the last month, have you often been bothered by feeling down, depressed or hopeless?  Yes □ No □  During the last month, have you often been bothered by having little interest or pleasure in doing things?  Yes □ No □  Is this something with which you would like help? No □ Yes but not today □ Yes □  From Hospital Anxiety and Depression questionnaire: Anxiety: Depression:  Pain catastrophising score: | | |
| **Family History:**  Anyone in the family with facial pain: No □ Yes □ Who  Anyone in the family with depression:  Anyone in the family with migraine:  *Father* Alive □ Deceased □ Health:  *Mother* Alive □ Deceased □ Health: *Siblings:* Number male female Health: | | |
| Social History and the effect the pain has on it: School & childhood  Relationship with parents/siblings/other family  Work and satisfaction  Marital status  Children  Finance  Housing  Other life events  Sources of stress  Leisure activity/relaxation activities  Significant life events: Yes □ No □ Unsure □ | | |

**Facies:** skin □ ears □ nose □ eyes □

**Swellings:** neck nodes □ weight □ BP □ pulse □

**Cranial nerves examination:**

| 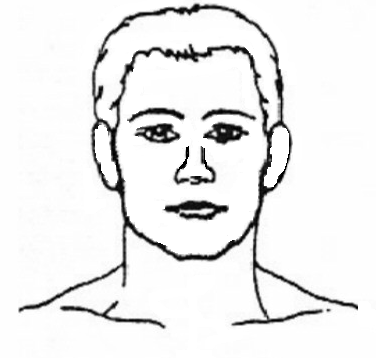 |  | 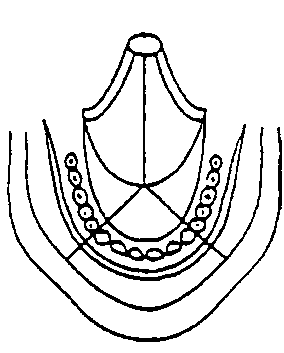 |
| --- | --- | --- |
| **Test** | **Right** | **Left** |
| Corneal reflex | Present/reduced/absent | Present/reduced/absent |
| Sensation Va | Normal/altered/numb/allodynia | Normal/altered/numb/allodynia |
| Sensation Vb | Normal/altered/numb/allodynia | Normal/altered/numb/allodynia |
| Sensation Vc | Normal/altered/numb/allodynia | Normal/altered/numb/allodynia |
| Other cranial nerves |  |  |
| Muscle tenderness/trigger points | Temporalis/masseter/  medial pterygoid/lateral pterygoid  /sternocleidomastoid/trapezius | Temporalis/masseter/  Medial pterygoid/lateral pterygoid sternocleidomastoid/trapezius |
| TMJ joint sounds Clicking | Soft loud consistent intermittent  Cycle early mid late  Painful painless  Opening Closing both | Soft loud consistent intermittent  Cycle early mid late  Painful painless  Opening Closing both |
| TMJ joint sounds Crepitus | painful painless | painful painless |
| If patient has consistent clicks, do these clicks disappear when the patient opens from a protrusive mandibular position? Yes no | | |
| Mouth opening | Normal Limited ( )mm opening  Lateral  Vertical comfortable maximal | |
| Type of occlusion | Class I Class II div i Class II div ii class III open bite | |
| Wear facets □ | Attrition # teeth | Keratosis tongue scalloping |

**Oral Hygiene:** Good □ Moderate □ Poor □ Periodontal disease □

**Dentition:** No teeth □ Partial dentition □ Full dentition □ Dentures □

**Conservation:** Nil □ Little □ Moderate □ Extensive □ Caries □

| **INVESTIGATIONS**  Xrays □ computed tomography (CT) SCAN □  Magnetic resonance imaging (MRI) □ neurophysiology □  hemoglobin & full blood count□ erythrocyte sedimentation rate □ c-reactive protein □ Folate □ B12 □  urea & electrolytes □ liver function test □ gamma-glutamyl transferase □ glucose □ thyroid function □ immunological □ others □ | | |
| --- | --- | --- |
| **PROVISIONAL DIAGNOSIS:** possible □ probable □ definite □ **primary** 1^0^ or **secondary** 2^0^  Dental related pain □  Temporomandibular disorder (TMD) with/without disc involvement □ Chronic facial pain □  Post traumatic stress □  Trigeminal neuralgia □ Atypical trigeminal neuralgia □   \| Neuropathic pain □ \| Atypical odontalgia □ \| Burning mouth syndrome/dysaesthesia □ \| \| --- \| --- \| --- \| \| Migraine □  Other:  Tension headache  **Co morbidities:**  Possible □  Depression □  Eating disorders □  Obsessive compulsive disorder □ \| Cluster headache □  Short-lasting Unilateral Neuralgiform headache attacks with Conjunctival injection and Tearing (SUNCT) / short-lasting unilateral neuralgiform headache attacks with cranial autonomic symptoms (SUNA) □  Probable □ Definite □  Anxiety □  Somatic concerns □  Chronic pain elsewhere □ \| Paroxysmal hemicrania □  Hemicrania continua □  Medically unexplained symptoms □  Adjustment reaction □  Hypochondriacal beliefs □  Hyper- vigilant □ Other \| | | |
| **MANAGEMENT**: Reassurance □  1. Information prescription: skull model PowerPoint other  2. resources: *pain toolkit, flyer “Manage your Pain”, flyer “Overcoming Chronic Pain”, Pain management plan, trigeminal neuralgia, facial pain, Burning mouth syndrome, TMD, psychology, mutidiciplinary team (MDT), online resources* | | |
| **2. Medication recommended** | **Daily dose** | **Frequency** |
|  |  |  |
|  |  |  |
|  |  |  |
|  |  |  |
| Patient did not want medication □  3. Pain diary □ ReliefInsite.com 4. Drug dosage schedule □  5. Contact with: support group □ Trigeminal neuralgia Association □ OUCH charity □  Expert patient program □  6. Physical Exercises □ 7. Relaxation □  **8. Other:** | | |
| **11. Referral** dental □ psychiatry □ MDT: neurology □ neurosurgery □ physio □  Psychology/pain management □ complimentary and alternative medicine □ Hypnotherapy □ Acupuncture □  **Clinicians estimate of compliance**: Excellent □ V. Good □ Good □ Fair □ Poor □  **REVIEW:** None □ Weeks _ Months _ (details: )  **SIGNATURE:**  Consultant/Specialty registrar/Specialty doctor/other  **PRINT NAME:** | | |

Supplementary material 2

**FACIAL PAIN—FOLLOW-UP**

| **DATE: CONSULTANT:** Seen: on their own/accompanied/advocate/language line ref no | **SURNAME: Male/Female**  **OTHER NAME:**  **Date of birth:** | | |
| --- | --- | --- | --- |
| **History since last visit:** | **Hospital number:** | | |
| **Diagnosis last visit:**  **Results of investigations:**  **How are you coping with your pain now: no better** □ **better** □ **worse** □ | | | |
| **Frequency of pain now:** continuous □ intermittent □ daily □ weekly □ monthly □  **Number attacks daily:**  **Change from last visit:** better □ worse □ no change □  **Pattern of pain now (draw diagram):**  **Periods of complete pain remission:** Yes □ No □ with drugs □ without drugs □  **Provoking factors:** | | | |
| **Severity/Intensity: worse/same/better** | | | |
| **Site: Same/different**  **Side: Right**□ Left□ Bilateral □ Trigeminal nerve branches: Va □ Vb □ Vc □ beyond V □ | | | |
| \| **Character:**  Cramping  Gnawing  Aching  Heavy \| \| --- \| \| Throbbing  Tender  Nagging  Other  Tiring  Frightful  Punishing  Unbearable  Burning  Hot  Pins and needles  Shooting  Stabbing  Sharp  Splitting \| | | | |
| **Medication: able to take** □ **took regularly** □ **took for: irregular** □  **Did not take (reasons)** □ | | | |
| **DRUGS SINCE LAST VISIT** | **DAILY DOSE** | **EFFECTIVENESS** | |
|  |  |  | |
|  |  |  | |
|  |  |  | |
|  |  |  | |
| **Side effects from drugs: None**□  Rash □ Dry mouth □ Dizzy □ Drowsy/tired □ Disturbed vision □ Ataxia □  Unable to concentrate □ Feel like zombie □ Abnormal sensations □  Gastro-intestinal nausea □ Vomiting □ Constipation □ Diarrhoea □  Other: Adverse event profile: yes/no | | | |
| **How disabling are the side effects: Mild** □ **Moderate** □ **Severe** □ | | | |
| **Other treatments:**  - lifestyle  - psychological support  - relaxation techniques | | | |
| **Hospital Anxiety and Depression scale:**  Anxiety score = Depression score =  **Brief Pain Inventory scale:** Yes □ No □ | | | |
| **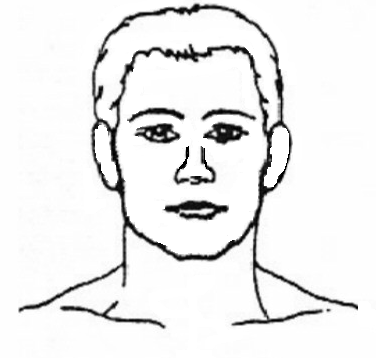**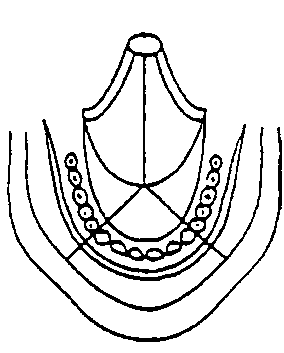  **Clinical examination (note any changes) Tenderness** □ **Limitation of opening** □  **Cranial nerve: trigeminal nerve - RVa** □ **RVb** □ **RVc** □ **LVa** □ **LVb** □ **LVc** □ | | | |
| **INVESTIGATIONS:**  Xrays □ computed tomography (CT) SCAN □  Magnetic resonance imaging (MRI) □ neurophysiology □  hemoglobin & full blood count□ erythrocyte sedimentation rate □ c-reactive protein □ Folate □ B12 □  urea & electrolytes □ liver function test □ gamma-glutamyl transferase □ glucose □ thyroid function □ | | | |
| **DIAGNOSIS:** probable □ or definite □  Dental □  Temporomandibular disorder (TMD) with/without disc involvement □ Chronic facial pain □  Trigeminal neuralgia (TN) □ TN with concomitant pain □ Secondary TN □   \| Neuropathic pain □ \| Persistent dentoalveolar pain disorder □ \| Burning mouth syndrome □ \| \| --- \| --- \| --- \| \| Migraine with/without aura □  Hemicrania continua □  Other:  **Co morbid**  Depression □  Eating disorders□  Obsessive compulsive disorder □ \| Short-lasting Unilateral Neuralgiform headache attacks with Conjunctival injection and Tearing (SUNCT) / short-lasting unilateral neuralgiform headache attacks with cranial autonomic symptoms (SUNA) □  Probable □  Anxiety □  Somatic concerns □  Chronic pain elsewhere □ \| Paroxysmal hemicrania □  Definite □  Adjustment reaction □  Hyper vigilance □  Post traumatic stress □ \| | | | |
| **MANAGEMENT:**  **1. Education/Addressing concerns setting goals**  **2. Other resources:** | | | |
| **3. Medication** | **DOSE range/sliding scale** | **FREQUENCY** |  |
|  |  |  |  |
|  |  |  |  |
| **4. Pain diary:** Yes □ No □  **5. Dosage schedule:** No □ Yes □ | | |  |
| **6. Referral**  **dental □ psychiatry □ Multidisciplinary team: neurology □ neurosurgery □**  **complementary and alternative medicine □**  **Psychology/pain management □**  **REVIEW:**  **None □ Weeks □ Months □ if required□ Database □ Consent to contact □**  **SIGNATURE:**  Consultant/Specialty registrar/Specialty doctor/other  **PRINT NAME:** | | | |
|  | | | |
